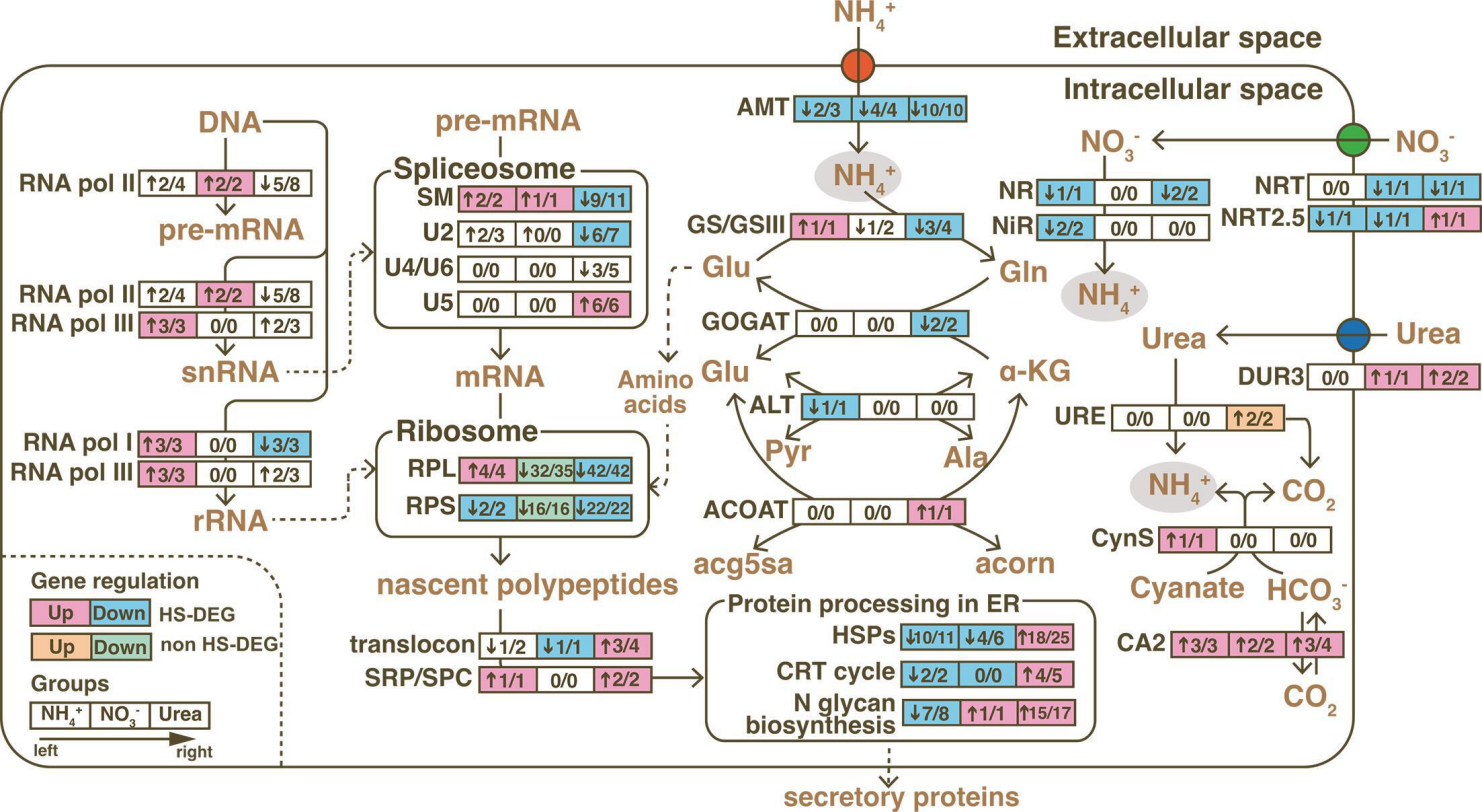# Articles of Significant Interest in This Issue

**DOI:** 10.1128/aem.00286-25

**Published:** 2025-02-19

## A METAGENOMIC VIEW OF CYANOBACTERIAL BLOOMS FROM THE AFRICAN CONTINENT

Cyanobacterial harmful algal blooms (cyanoHABs) threaten freshwater systems globally. Hart et al. (e01507-24) used metagenomics to describe the dynamics and toxin biosynthetic potential of cyanoHABs in the Winam Gulf of Kenya. This information is important for interventions that mitigate the harmful blooms.


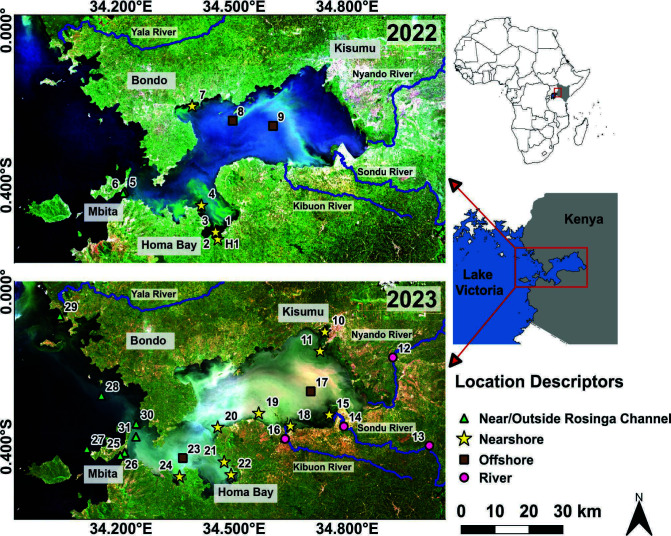


## A QUORUM PATH TO CORAL PATHOGENESIS

Lydick et al. (e01143-24) describe the quorum-sensing pathways of several strains of *Vibrio coralliilyticus*, an emerging coral pathogen. This information can guide therapeutic efforts to alter disease in corals.


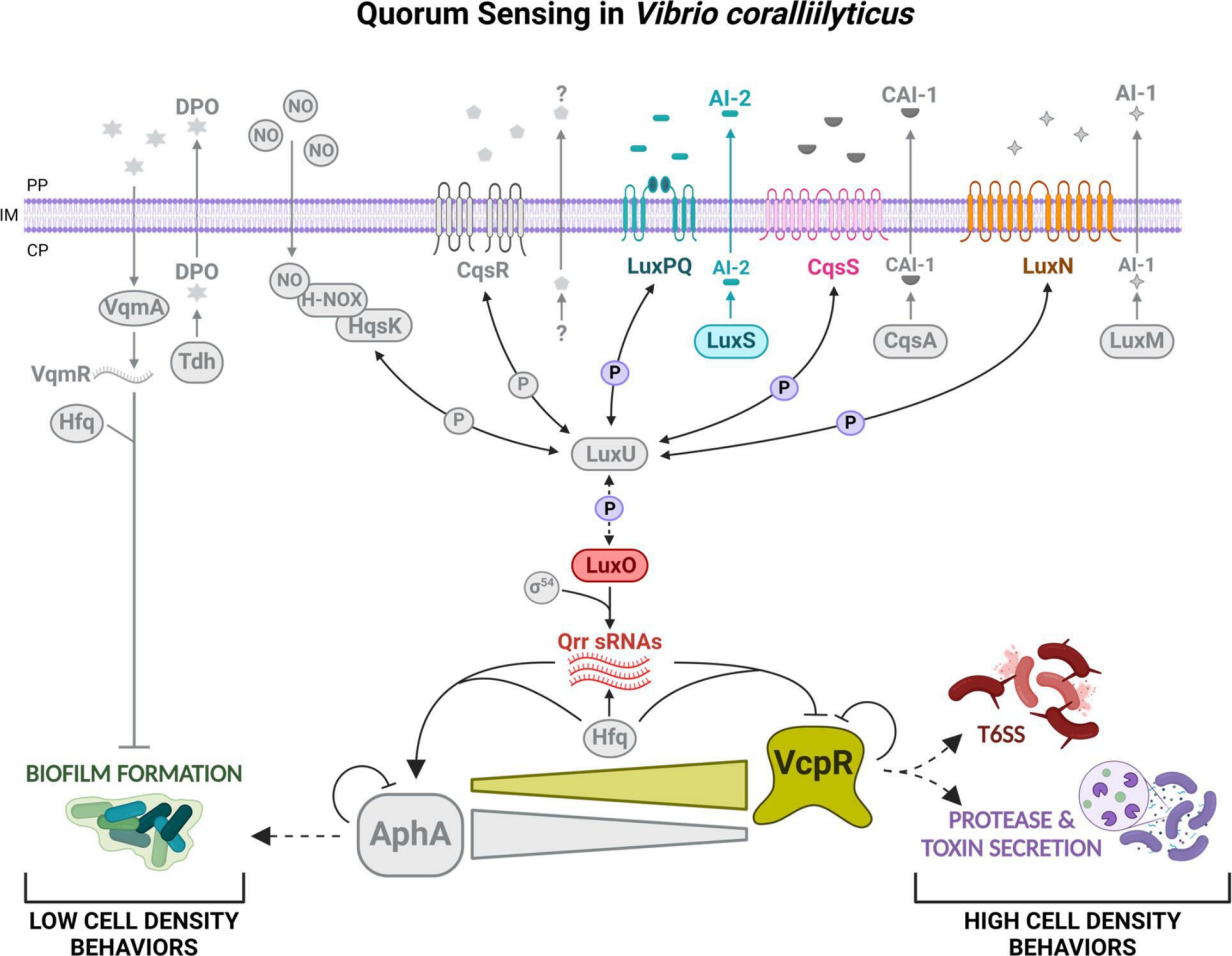


## A *WOLBACHIA* INTERVENTION FOR TIGER MOSQUITO CONTROL

The tiger mosquito is a highly invasive and efficient vector for human arboviruses such as Dengue and Chikungunya. Lejarre et al. (e02350-24) describe an improved method for artificial infection with *Wolbachia* bacteria that renders the mosquito females sterile to prevent viral transmission.


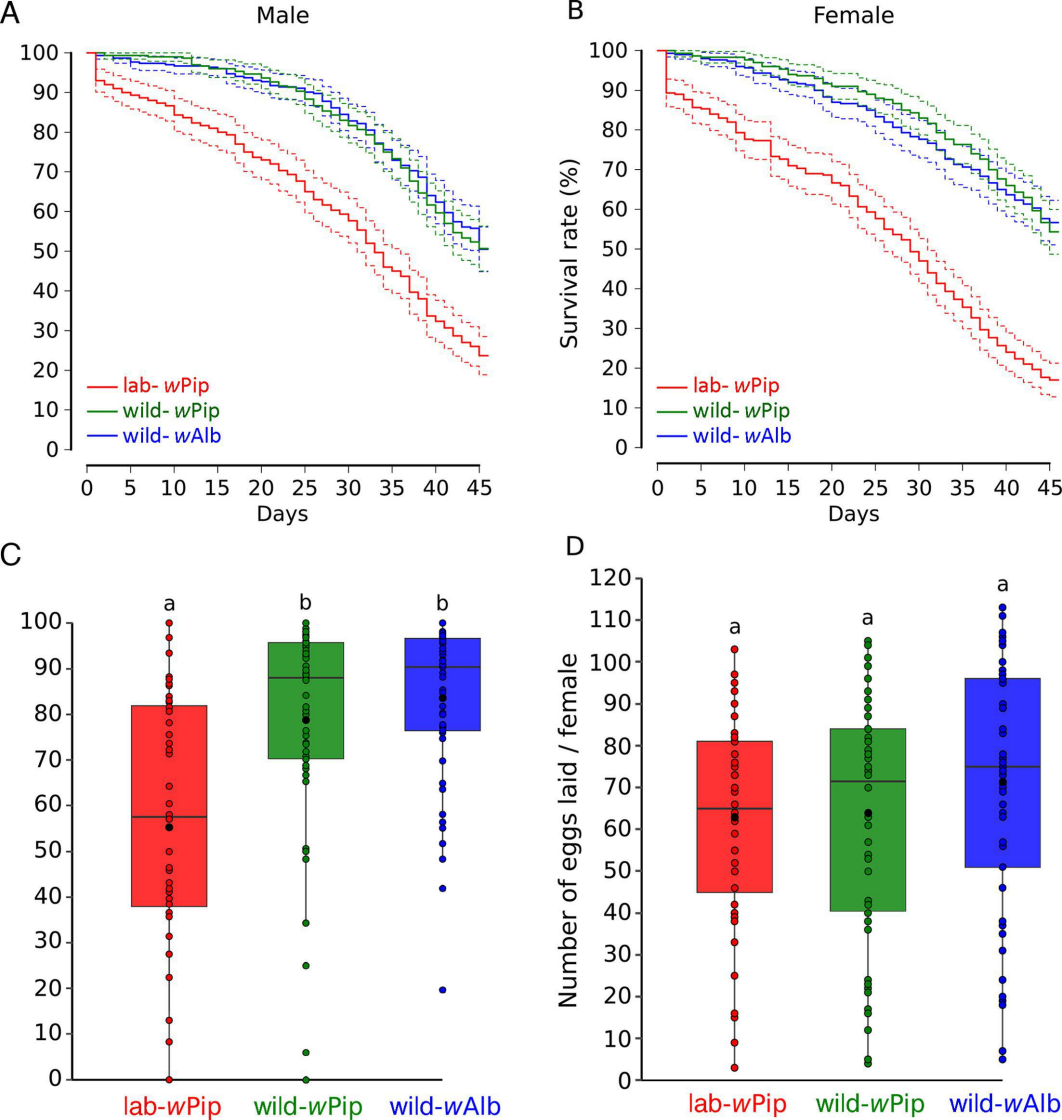


## BACTERIAL CELLULOSE PRODUCTION GETS CRISPR’ED

Xin et al. (e02455-24) describe a CRISPR approach for multiplex genome editing of cellulose-producing *Komagataeibacter* bacteria. This is an improvement over targeted mutagenesis via homologous recombination methods that can accelerate applications for bacterial cellulose.


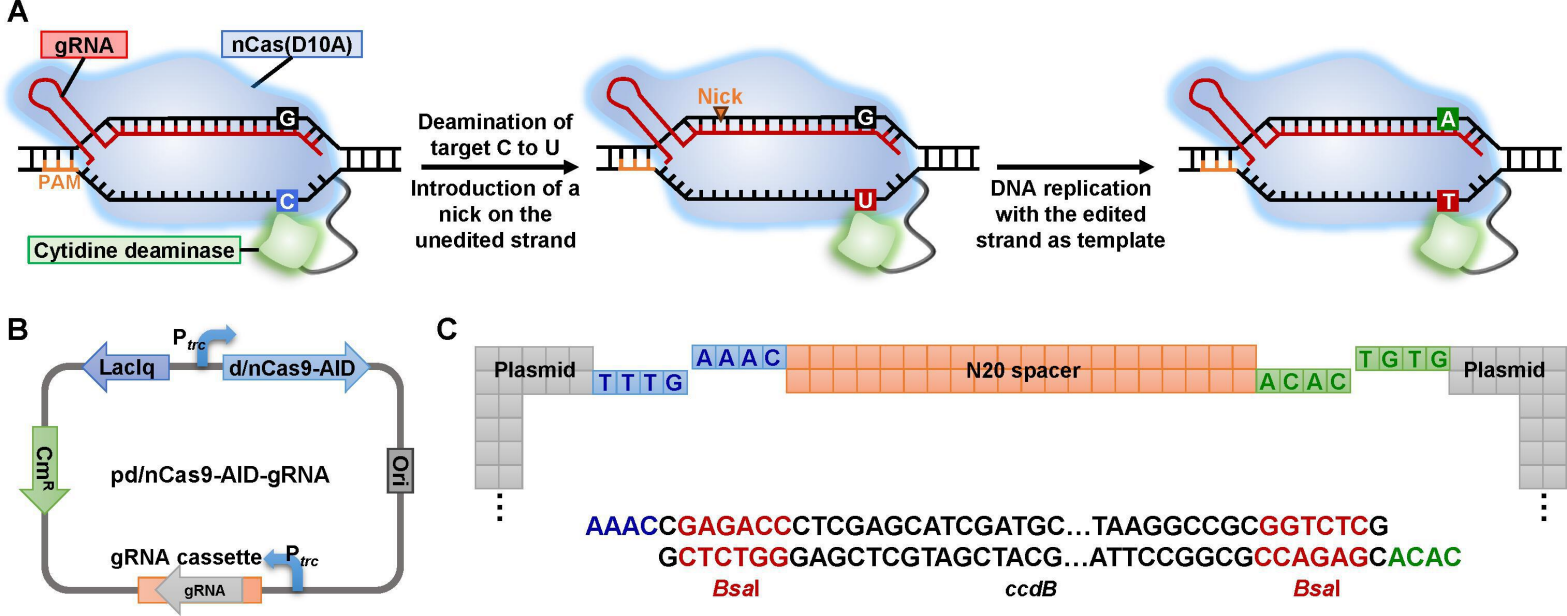


## MICROBES UNDER PRESSURE

A large yet relatively understudied diversity of microbes live under high hydrostatic pressures at the bottom of the oceans and within the subsurface of the Earth’s crust. Foustoukos and Houghton (e02010-24) review recent technical developments for their laboratory cultivation and study.


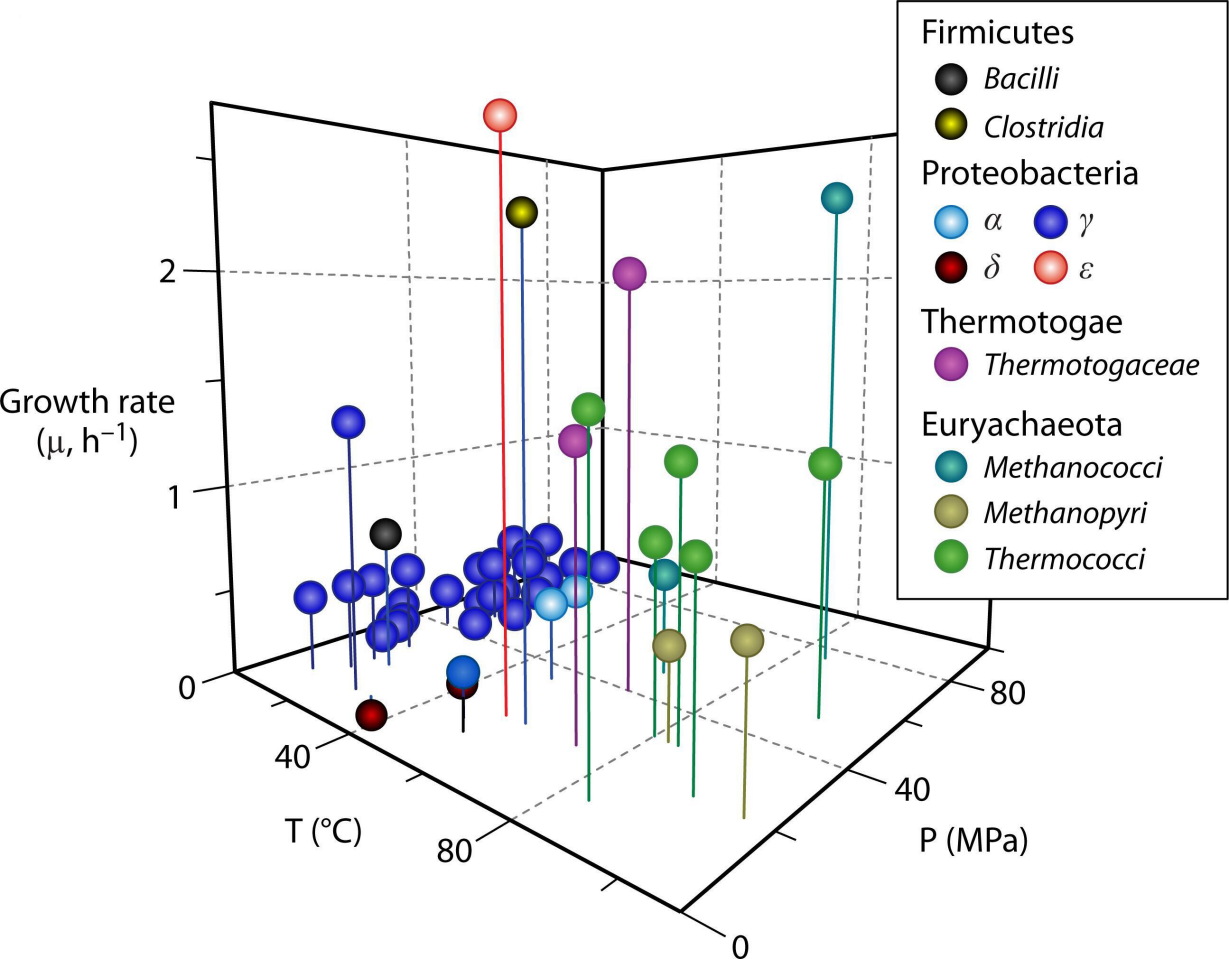


## THE AROMATIC FLAVOR OF BACTERIAL TRANSPORT

Meng et al. (e00827-24) describe a novel bacterial transporter for cross-membrane uptake of polycyclic aromatic hydrocarbons (PAHs). This is an important study to understand how hydrophobic compounds enter bacterial cells and enhance the bioremediation capacity of PAH-degrading bacteria.


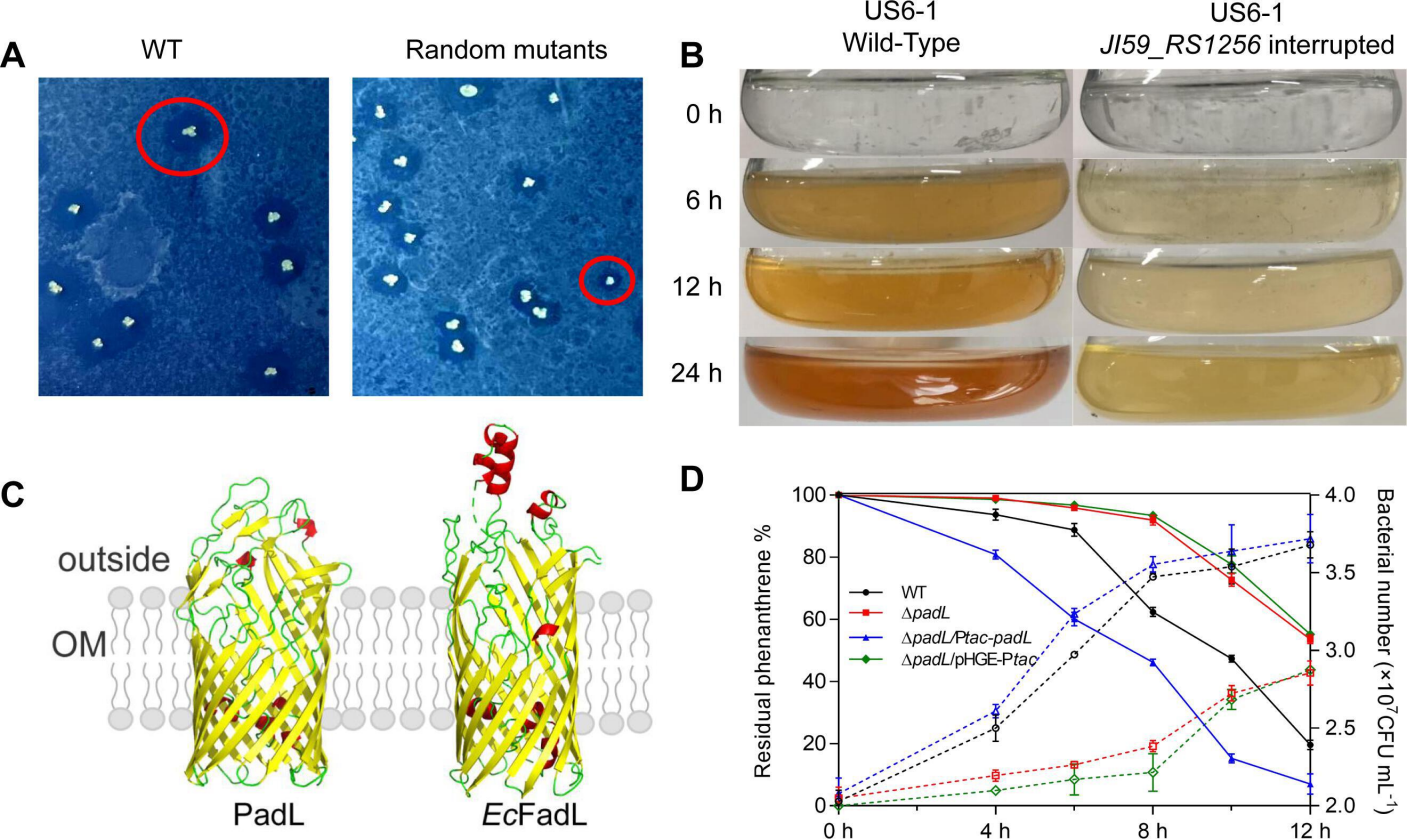


## THE NITROGEN DEPENDENCE OF CORAL BLEACHING

Huang et al. (e00591-24) describe the differential effects of nitrogen types on the heat stress response of a coral symbiont. These findings highlight the need to consider the type of nitrogen pollution, particularly urea, in coral conservation.